# The Functions of Atrial Strands Interdigitating with and Penetrating into Sinoatrial Node: A Theoretical Study of the Problem

**DOI:** 10.1371/journal.pone.0118623

**Published:** 2015-03-24

**Authors:** Xiaodong Huang, Xiaohua Cui

**Affiliations:** 1 Department of Physics, South China University of Technology, Guangzhou, China; 2 School of Systems Science, Beijing Normal University, Beijing, China; The Ohio State University, UNITED STATES

## Abstract

The sinoatrial node (SAN)-atrium system is closely involved with the activity of heart beating. The impulse propagation and phase-locking behaviors of this system are of theoretical interest. Some experiments have revealed that atrial strands (ASs) interdigitate with and penetrate into the SAN, whereby the SAN-atrium system works as a complex network. In this study, the functions of ASs are numerically investigated using realistic cardiac models. The results indicate that the ASs penetrating into the central region of the SAN play a major role in propagating excitation into the atrium. This is because the threshold SAN-AS coupling for an AS to function as an alternative path for propagation is lower at the center than at the periphery. However, ASs penetrating into the peripheral region have a great effect in terms of enlarging the 1:1 entrainment range of the SAN because the automaticity of the SAN is evidently reduced by ASs. Moreover, an analytical formula for approximating the enlargement of the 1:1 range is derived.

## Introduction

The sinoatrial node (SAN) is a small and thin sheet of tissue embedded within the right atrium and is surrounded by atrial cells. During heart beating, the impulses are initiated in the SAN and then propagate into the neighboring atrial cells. How the small SAN drives a large number of adjacent atrial cells and how the atrial cells influence the oscillating behaviors of the SAN are problems of interest in cardiovascular research. However, clear interpretations of these phenomena have not yet been obtained.

Many authors have investigated how the small SAN can drive the atrium. First, the self-oscillatory SAN cells must fire synchronously. This synchronization can be achieved by the currents flowing through the gap junctions between neighboring cells [1, 2]. Second, the peripheral SAN cells must overcome the hyperpolarization from the atrium to fire. It is known that the atrial cells have a resting potential of approximately −80 mV, which is lower than the maximum diastolic potential of SAN cells (approximately −60 mV). Therefore, it is theoretically very difficult for the small SAN to drive the atrium because of hyperpolarization. Joyner and van Capelle [3] investigated how conduction could be guaranteed. These authors concluded that a gradient distribution of junctional conductance in the SAN (decreasing from the center to the periphery) is essential. However, ten Velde *et al*. [4] experimentally found that a gradient distribution of coupling does not actually exist. They used immunofluorescence to visualize the myocyte distribution in the SAN and atrial tissue. Based on their results, they hypothesized that there are strands of atrial cells that interdigitate with and penetrate into the SAN tissue, which are coupled with the SAN cells. Therefore, the atrial strands may serve as alternative paths for impulse propagation. They concluded that the pacemaking of SAN is shielded from atrium hyperpolarization by tissue geometric factors rather than by gradient coupling between SAN cells. The observations of Ref. [5] also partly supported this conclusion. Winslow [6–8] substantiated the function of the atrial strands in impulse propagation in a numerical model. Recently, the pacing and impulse propagation in realistic heterogeneous SAN model is studied by Oren and Clancy [9]. In their simulations, the influences of SAN cell types, coupling gradient, atrial load and fibroblasts on pacing and propagating dynamics are investigated in detail. The study well approaches the practical situations of integral SAN-atrium tissue. Given the above results, by incorporating cell type heterogeneity and geometry complexity into the model, some novel mechanistic insights of the problem may be obtained. Thus, as one aspect of this work, we will investigate how the conduction depends on the SAN-atrium coupling strength and whether the coupling positions in SAN tissue may influence the efficiency of the atrial strand.

How the atrial cells influence the phase-locking behaviors of the SAN is another interesting problem. Phase-locking can be simply defined in a system consisting of two coupled periodic oscillators (or one nonlinear oscillator influenced by periodic external stimulations) as the frequency entrainment or phase entrainment on the Poincare section. It is an important issue in the basic research of nonlinear dynamics, which reveals the general mechanisms of inducing synchronization, aperiodic and chaotic behaviors in nonlinear systems [10–12]. Phase-locking theories may apply to disciplines such as electronics (e.g., circuit oscillation), complex networks and system biology (e.g., neurology, cardiology). In particular, SAN is an important type of biological oscillator. The rhythm of SAN is adaptive to different needs of body, i.e., its oscillating frequency can be modulated by external factors. Therefore, phase-locking behaviors of SAN may imply rich rhythmic patterns in heart beating [13–17]. The study of SAN phase-locking may help understanding the synchronous firing, parasystole and heart rate regulations in practice. It may also enrich the basic theories of nonlinear dynamics. This is another motivation of the present study.

Many authors have applied phase-locking behaviors to investigate the diverse rhythmic oscillations of biological systems [18–21]. Theoretical and experimental studies have shown diverse phase-locking behaviors in realistic biological oscillators [22–29]. To explain the phase-locking behaviors of the SAN and other biological oscillators, Winfree [18] and some other authors [19, 20, 24, 28, 30–32] developed the phase resetting theory, which has yielded great success. However, in previous studies, SAN dynamics was usually investigated using simple systems (e.g., a single cell or oscillator [19, 20, 23] or homogeneous tissue [33]). In fact, SAN tissue constitutes a heterogeneous system [34, 35], which may exhibit some interesting phase-locking behaviors [36]. Furthermore, in the presence of the geometric complexity brought by atrial strands, the phase-locking dynamics may show some new characteristics, which better approach the actual dynamics in the heart realistically. Thus, we are interested in the phase-locking behaviors of the complex SAN-atrium system, which forms another aspect of the present work.

In the present paper, a heterogeneous SAN tissue model [37] with homogeneous coupling is used. In such a system, the functions of atrial strands in impulse propagation and phase-locking behaviors are investigated. The major results of the present work are as follows: (i) coupling conditions for successful pacemaking and driving are found, and the threshold coupling strength for an atrial strand to become a useful path for propagation is determined. (ii) In the presence of atrial strands, the 1:1 phase-locking range of the SAN could be effectively enlarged, while the other phase-locking ranges are almost unchanged. An analytical formula for approximating the enlargement of the 1:1 range is derived. Our work may provide novel insights into the basic research of nonlinear dynamics and could also be applicable in practical cardiovascular research.

## Methods

**Fig 1:**
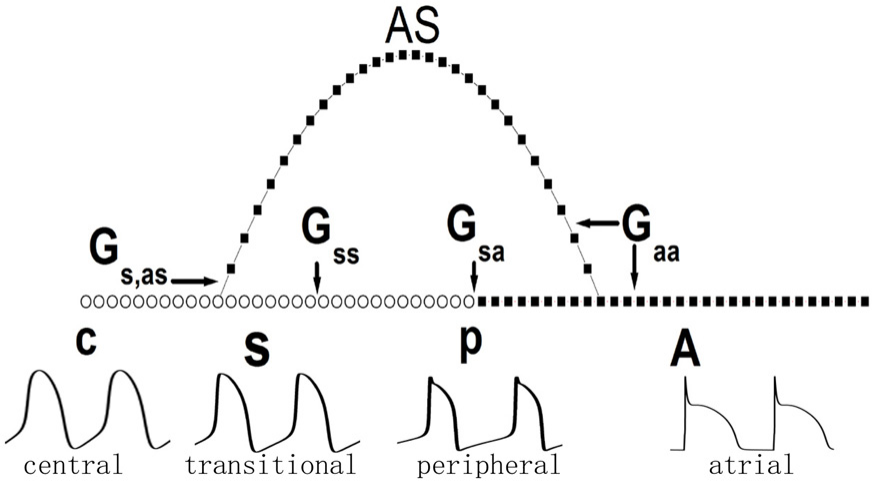
Geometry of the model. Empty circles labeled “**S**” represent the SAN, and solid squares labeled “**A**” represent the atrium. The letters “c” and “p” denote the central and peripheral ends of the SAN, respectively. The atrial strand (labeled “AS”) links a SAN cell and an atrial cell by its ends. Atrial cells in the AS are identical to those in the atrium. The AS is identified as (*x*-*y*), with *x* and *y* being the cells at distances *x* and *y*, respectively, relative to the left-most end of the cable. The couplings between different cells are indicated in the corresponding areas. The action potentials of the typical cells from left to right are illustrated in the bottom trace.

The SAN-atrium system is a 3-dimensional structure. As hypothesized and observed by ten Velde et al. [4] and Dobrzynski et al. [5], the connections between SAN and atrium are very complex. The atrial cells penetrate like bundles into the SAN region and interdigitate with SAN cells. In this sense, the SAN-atrium system is a complex network in topology. In our physical study, we would like to simplify the geometry to reveal the basic properties of the system. Viewing from the cross section of SAN-atrium tissue (as shown by Fig.4 in Ref. [5]), the atrial bundles may look like bridges that link the atrium and SAN. The excitation from SAN may propagate via the atrial tissue directly coupled on the periphery and the bridge like atrial bundles. With respect to the pacing and propagating dynamics of the SAN, the SAN-atrium tissue can be simplified as a 1-D cable with atrial strands linking the SAN and atrial tissue at both of its ends. Fig. 1 illustrates the geometry of the model. In the cable, the left region indicated by empty circles and labeled “S” represents the SAN, and the right region of solid squares and labeled “A” represents the atrium. The chain consisting of atrial cells labeled “AS” represents the atrial strand. The number of ASs and the positions of the linkage can be varied. We use the positions of the linked cell pair to identify an AS, which is represented as (*x*-*y*), where *x* and *y* denote the cells at distances *x* and *y*, respectively, as measured from the left-most end of the cable. The strengths of the coupling between different cells are represented by *G*_*ss*_ (SAN-SAN cell coupling), *G*_*sa*_ (SAN-atrial cell coupling), *G*_*s*,*as*_ (SAN-AS cell coupling), and *G*_*aa*_ (atrial-atrial cell coupling), which are indicated in the corresponding areas in Fig. 1.

For SAN tissue, the heterogeneous rabbit SAN model [36, 37] is used. There are some other models describing more details of physiological processes in SAN cell (e.g., calcium cycling) [38]. In the present model, the basic differential equation for the dynamics of the membrane voltage *V* is

$$\begin{array}{c} C_{m}\left(x\right)\frac{\partial V(x,t)}{\partial t}=-I_{ion}\left(V,y\right)+D\nabla^{2}V\left(x,t\right)+I_{st}\left(x,t\right). \end{array}$$ (1)

*I*_*st*_(*x*, *t*) denotes the stimulating current, which is delivered to the *x*th cell if it is used. The total ionic current *I*_*ion*_ consists of 13 individual currents. Each ionic current is a function of *V* and gating variables (represented by vector **y**). Each gating variable evolves as

$$\begin{array}{c} \frac{dy}{dt}=\frac{y_{\infty }-y}{\tau_{y}}, \end{array}$$ (2)

where *y* represents any gating variable and *τ*_*y*_ and *y*_∞_ are the time constant and the steady state of *y*, respectively. As indicated in Ref. [37], from the center to the periphery, the capacitances of the SAN cells range from 20 pF to 65 pF, and the ionic conductances vary correspondingly. According to experimental observations, Zhang *et al*. [37] treated the capacitance and conductance of the tissue as functions of the distance *x* from the central end. The heterogeneity of SAN tissue is represented using the following functions: the capacitance of the cell at *x* is

$$\begin{array}{c} C_{m}\left(x\right)=20+\frac{1.07(x-0.1)}{L[1+0.7745e^{-(x-2.05)/0.295}]}\left(65-20\right), \end{array}$$ (3)

and the conductance of any current on this cell is

$$\begin{array}{c} g_{z}\left(x\right)=\frac{\left[65-C_{m}\left(x\right)\right]g_{zc}+\left[C_{m}\left(x\right)-20\right]g_{zp}}{65-20}. \end{array}$$ (4)

Note that *L* is the total length of the tissue (*L* = 30×0.1 mm = 3 mm in our work), and *g*_*zc*_ and *g*_*zp*_ represent the conductances of the ionic current *z* of the central end and peripheral end, respectively. Details of the model and the set of the parameters appear in Refs. [36, 37]. The action potentials of the typical cells within the heterogeneous SAN tissue are illustrated in the bottom trace of Fig. 1.

As for the atrial tissue, there are well developed models for it [39, 40]. The basic physical property of an atrial cell is excitability, as is that of the ventricular cell. Therefore, the well-developed ventricular models are often used to simulate atrial cells [3, 41]. In the present work, the simple model that describes guinea pig ventricular cells, the LR1 model [42], is used to describe the atrial cell. The dynamics of the atrial tissue (both the atrium and AS) also follow Eqs. (1) and (2), with different compositions of *I*_*ion*_ and parameter values corresponding to the LR1 model. The parameters *G*_*si*_ and *G*_*K*_ are modified to be 0.035 mS/cm^2^ and 0.705 mS/cm^2^, respectively. The membrane capacitance *C*_*m*_ is 1 *μF*/cm^2^. In such a parameter set, the data of the action potential simulate the experimental results during pacing of SAN. A detailed description of this model is presented in Refs. [42, 43]. The action potentials of the modeled atrial cell are shown in the bottom trace of Fig. 1, labeled as “atrial”.

For the numerical simulations, the explicit Euler method is used. The spatial resolution is taken to be 0.032 cm for the LR1 model and 0.01 cm for the SAN model (just as that used in Ref. [37]). The time step is 0.02 ms. The gating variables are integrated using the method developed by Moore and Ramon [44] and Rush and Larsen [45]. No flux boundary condition is used. In the atrial tissue and ASs, the coefficient *D* is fixed to be 1.25 cm^2^/s (providing a conduction velocity of approximately 60 cm/s). In the present work, *G*_*ss*_, *G*_*sa*_, and *G*_*s*,*as*_ are varied (corresponding to the variations of diffusion coefficient *D* in Eq. (1)) to investigate the effects of the coupling on the system dynamics. The stimulating current *I*_*st*_(*x*, *t*), if used, is delivered to the cell at *x* and in pulsatile form with a duration of 2 ms and magnitude of 50 nA/nF. To measure entrainment, the first 500,000 steps are discarded, and the steady state is used.

## Results and Discussion

In this section, the functions of the atrial strands (ASs) are presented and discussed. The problems of impulse propagation and phase-locking behaviors in such a system are studied in the following subsections.

### The function of atrial strands in impulse propagation

**Fig 2:**
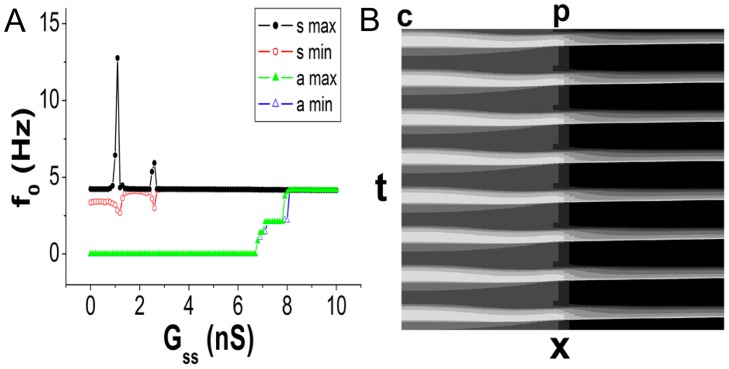
Synchronization and pacing ability of the system without an AS. A: The maximum and minimum frequencies of the SAN (the circles) and atrial tissue (the triangles) as functions of *G*_*ss*_ (*G*_*sa*_ = 20 nS). When *G*_*ss*_ ≥ 3.8 nS, synchronization is achieved in the SAN. Between 6.8 nS and 8 nS, the atrium is paced but not 1:1 synchronized with the SAN. When *G*_*ss*_ > 8 nS, normal beating of the tissue is realized. B: The spatiotemporal pattern of the normal beating of the system (*G*_*ss*_ = 20 nS and *G*_*sa*_ = 20 nS). The abscissa labeled *x* indicates the positions of the cells, and the ordinate labeled *t* denotes the time. “c” and “p” denote the central and peripheral ends of the SAN, respectively. The membrane voltages are shown by colors ranging from black to white. The lighter the color is, the larger the value.

First, the synchronization and pacing ability of the system without ASs are estimated. When coupling is weak in the SAN, each cell oscillates with an individual frequency. The maximum and minimum firing frequencies of the cells are plotted in Fig. 2A as functions of *G*_*ss*_ (*G*_*sa*_ is fixed to be 20 nS). It is observed that *G*_*ss*_ = 3.8 nS is sufficient for synchronization of the SAN tissue (the traces of “s max” and “s min” coincide with each other when *G*_*ss*_ ≥ 3.8 nS). However, the SAN is unable to drive the atrium (the frequencies of the atrial cells are all 0) until *G*_*ss*_ ≥ 6.8 nS. The reason for this result is that the hyperpolarization from atrial cells is considerably heavier than the excitation from the neighboring SAN cells when *G*_*ss*_ is small, and thus, the peripheral SAN cells cannot fire and propagation is blocked in the peripheral region. Note that when 6.8 nS ≤ *G*_*ss*_ ≤ 8nS, the firing frequencies of the atrium are not 1:1 synchronized with the SAN. When *G*_*ss*_ > 8 nS, normal beating of the system is realized. Fig. 2B shows the spatiotemporal pattern of the normal beating (*G*_*ss*_ = 20 nS and *G*_*sa*_ = 20 nS).

**Fig 3:**
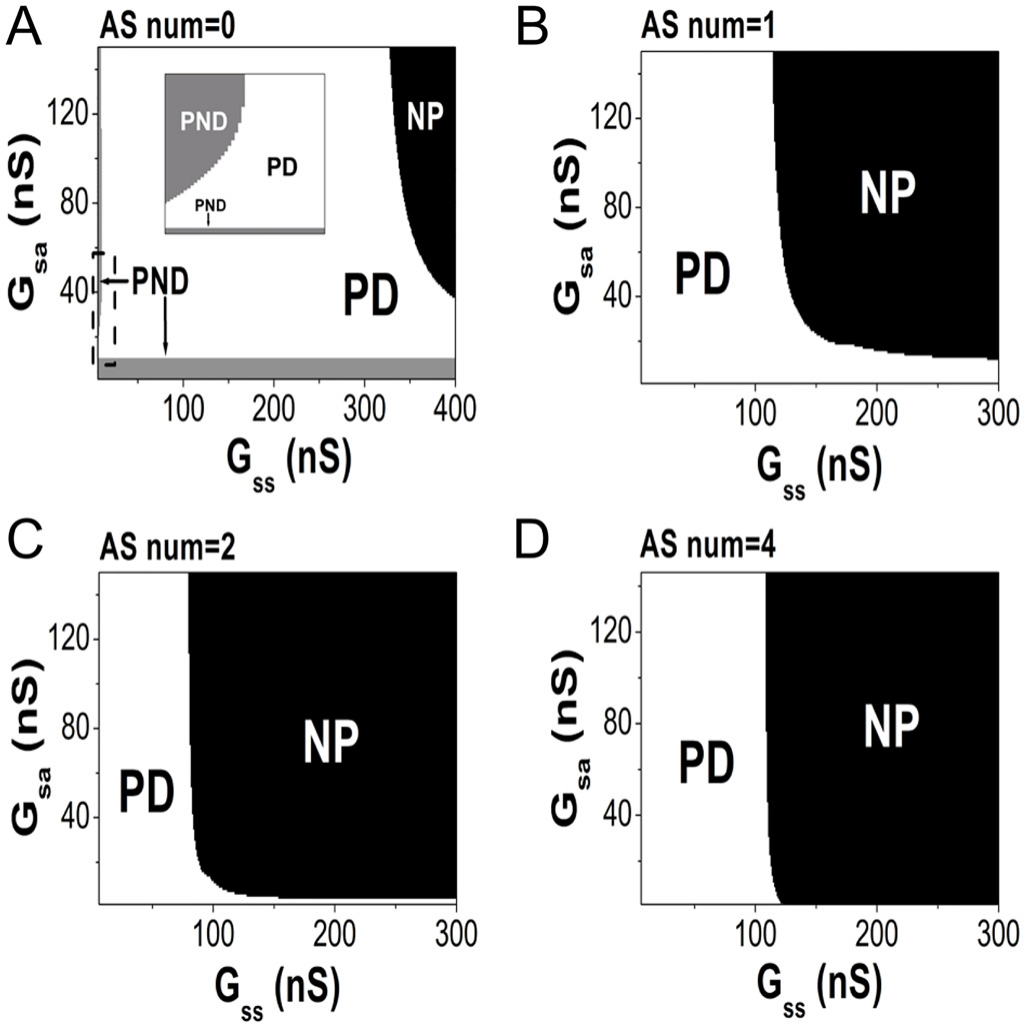
The parameter conditions for the pacing of the system. The parameters *G*_*ss*_ and *G*_*sa*_, which determine the pacing ability, are used to construct the parameter plane. *G*_*s*,*as*_ = 5 nS for all ASs if they exist. Notations **PND**, **PD** and **NP** denote the SAN behaviors. A: The situation without an AS. When either *G*_*ss*_ is small or *G*_*sa*_ is small, impulse propagation fails (see the gray regions labeled “PND”). The inset is a magnification of the dashed squared frame. If *G*_*ss*_ is very large, the pacing of the SAN fails (see the black region labeled “NP”). B: A single AS (11–32) is added to the system. C: Two ASs (11–32) and (23–34) are added. D: Four ASs (17–32), (20–34), (23,36) and (26,38) are added. In all cases with AS(s), the PND regions are completely eliminated.

The function of ASs in propagation can be investigated by examining the *G*_*sa*_-*G*_*ss*_ parameter plane, as shown in Fig. 3. In this figure, **PND** stands for “SAN **paces** but does **not drive** the atrium”, **PD** stands for “SAN **paces** and **drives** the atrium successfully”, and **NP** stands for “**no pacing** of SAN”. Fig. 3A shows the case of no ASs. Two distinct regions of PND, colored gray, appear when *G*_*sa*_ or *G*_*ss*_ is small. The inset shows the dashed squared frame, magnified. When *G*_*ss*_ is small and *G*_*sa*_ is sufficiently large, the peripheral SAN region will be too hyperpolarized to fire, and the vertical PND region forms. When *G*_*sa*_ is sufficiently small, the driving current from the SAN is too weak to excite the atrium, and thus, the horizontal PND region forms. If *G*_*ss*_ becomes very large, the entire system may fall into a quiescent state (see the black region labeled NP in Fig. 3A). This is because the SAN tissue is so tense that the hyperpolarization effect can easily impact the whole tissue. Figs. 3B-3D show the effects of AS(s) (the number of ASs is indicated on the top of each panel). For each AS, the coupling strength between its left end and the linked SAN cell is set to be 5 nS (i.e., *G*_*s*,*as*_ = 5 nS). The PND regions are completely eliminated in all cases with AS(s), which reveals that ASs can effectively propagate the impulse out of SAN even though direct propagation through the SAN periphery fails. However, the elimination of the PND regions occurs at the expense of enlargement of the NP region, which would be dangerous for the SAN. As the number of ASs increases, the NP region enlarges, which could occur because the ASs deliver extra hyperpolarizing currents to the SAN and make it easier for it to lose pacing ability. However, stopping pacing requires *G*_*ss*_ to be as high as 100 nS, which may not realistically occur in normal SAN tissue [4]. Therefore, the ASs play a positive role in impulse propagation in the heart.

**Fig 4:**
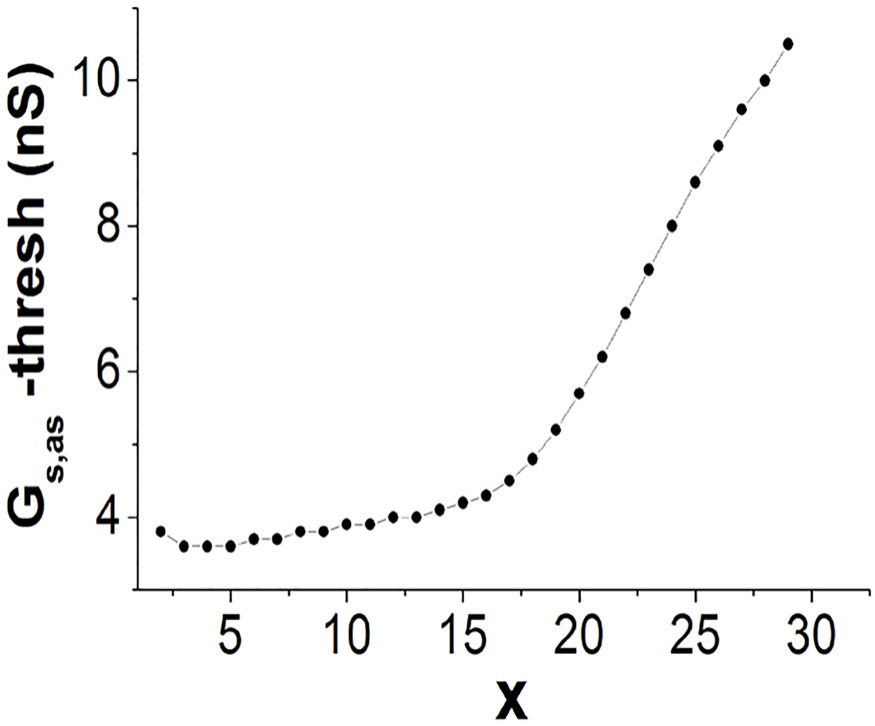
The minimum *G*_*s*,*as*_ for excitation of the AS (*x*-32) as a function of *x*. By setting *G*_*ss*_ = 20 nS and *G*_*ss*_ = 10 nS, the propagation from the SAN periphery to the atrium is hindered. From the center to the periphery, the threshold value increases. Therefore, with the AS penetrating into the central region, a useful path for propagation is more easily obtained.

We next investigate how large the *G*_*s*,*as*_ must be for an AS to become a useful path for propagation. We set *G*_*ss*_ = 20 nS and *G*_*sa*_ = 10 nS. This parameter set fails to achieve direct propagation via the SAN periphery. A single AS of (*x*-32) is investigated (*x* can be varied within the SAN). To conduct the impulse via the AS, the *x*th SAN cell must excite the coupled atrial cell on the AS end. Thus, there is a threshold value of *G*_*s*,*as*_, and when this value is exceeded, excitation occurs. The plot of the threshold value of *G*_*s*,*as*_ and the linking position *x* is shown in Fig. 4. By roughly dividing the SAN tissue into the central region (1 ≤ *x* ≤ 15) and the peripheral region (15 < *x* ≤ 30), it is observed that the threshold values of the central region are considerably smaller than those of the peripheral region. This result indicates that obtaining a useful path for propagation is achieved more easily with the AS penetrating into the central region of the SAN.

In this subsection, the functions of ASs in propagation are investigated. We found that even though propagation fails in the SAN periphery, successful driving of the atrium can still be realized. In particular, an AS penetrating into the central region, rather than into the periphery, is more likely to yield a useful path because of the lower threshold coupling strength involved, and we therefore conclude that ASs penetrating into the central SAN would positively impact driving the atrium.

### The function of atrial strands in phase-locking behaviors

The phase-locking behaviors of biological oscillators have been thoroughly studied. However, phase-locking in the SAN-atrium tissue with a complex geometry is seldom studied. In this subsection, the function of ASs in phase-locking is investigated, which may provide insights on the mechanisms of heart rate regulation.

**Fig 5:**
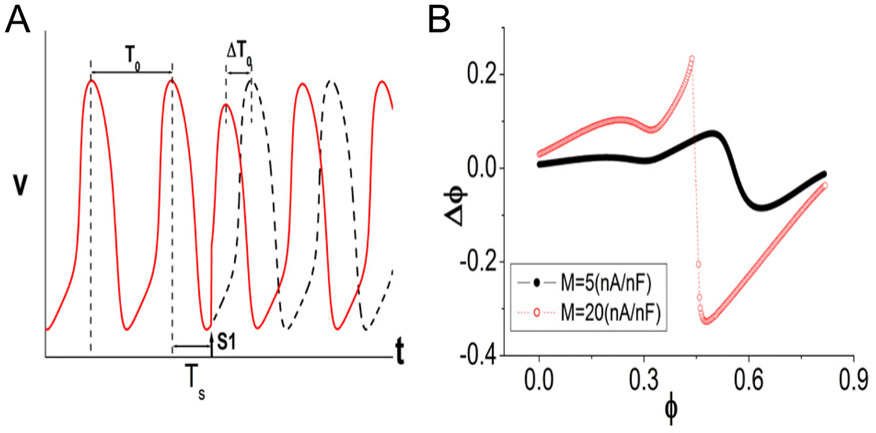
Phase resetting property of a single SAN cell. This figure was created using a numerical model [36]. A: Phase resetting of the action potentials. The dashed trace shows the unperturbed action potentials, and the solid trace represents that stimulated using one time period of stimulation (labeled S1). *T*_0_ is the intrinsic period of the unperturbed oscillations. *T*_*s*_ is the application time of S1, and Δ*T*_0_ is the time shift of the following action potential. By defining *ϕ* = *T*_*s*_/*T*_0_ and Δ*ϕ* = Δ*T*_0_/*T*_0_, it is found that Δ*ϕ* is a function of *ϕ*, which is the so-called phase resetting curve. B: The phase resetting curve. The shape of the curve is determined by the magnitude of S1. The curves corresponding to small (continuous) and large (discontinuous) magnitudes are shown.

First, we briefly introduce the methods for investigating phase-locking behaviors and describe the rotation number and phase resetting map (PRM). For the oscillations of the SAN cell, we define *T*_*i*_ as the time interval between the peaks of the *i*th and (*i* + 1)th action potentials. The rotation number can be written as

$$\begin{array}{c} \rho =\lim_{M\to \infty }\frac{MT_{sti}}{\sum_{i=1}^{M}T_{i}}, \end{array}$$ (5)

where *T*_*sti*_ is the stimulating period. A rational value for *ρ* implies a periodic state, whereas an irrational value for *ρ* implies quasi-periodic states or chaos. The theoretical approach to analyzing phase-locking dynamics involves the phase resetting map (PRM) [46], which is derived from the phase resetting property of the SAN. Fig. 5A is an illustration of the property of a single SAN cell. The dashed trace shows the unperturbed oscillations, and the solid line shows the oscillations perturbed by one period of stimulation (indicated by S1). It can be observed that the following action potentials after S1 are identical (or nearly identical) to the unperturbed ones, except for a shift of phase. We can define the phase of S1 as *ϕ* = *T*_*s*_/*T*_0_ and the phase shift of the following action potential as Δ*ϕ* = Δ*T*_0_/*T*_0_ (the quantities are indicated in Fig. 5A and explained in the caption). It is observed that Δ*ϕ* is a function of *ϕ*, which is called the phase resetting curve (see Fig. 5B). The shape of the curve depends on the magnitude of the stimulation [18, 27, 29]. During periodic stimulation with period *T*_*sti*_, the phases of successive *i*th and (*i* + 1)th stimuli satisfy [19, 20, 24]

$$\begin{array}{c} \phi_{i+1}=\phi_{i}-\Delta \phi_{i}+\frac{T_{sti}}{T_{0}}\left(\mathrm{mod}\;1\right), \end{array}$$ (6)

which is the so-called PRM. In this subsection, the rotation number *ρ* and PRM of Eq. (6) are used to analyze the results.

In our previous work, it was found that stimulations on different positions in the SAN cable result in different ranges of phase-locking [36]. Here, we focus on the comparison between the systems with and without ASs, so the stimulations are always delivered to the 29th SAN cell. Similar phenomena occur for other stimulated positions. In the present work, the stimulation is in pulsatile form with a duration of 2 ms and a magnitude of 50 nA/nF. *G*_*ss*_ = 20 nS, *G*_*sa*_ = 20 nS and *G*_*s*,*as*_ = 10 nS unless otherwise specified. Entrainment is estimated on the 10th SAN cell. As the stimulating period *T*_*sti*_ varies, phase-locking regions form the so-called “devil stairs”.

**Fig 6:**
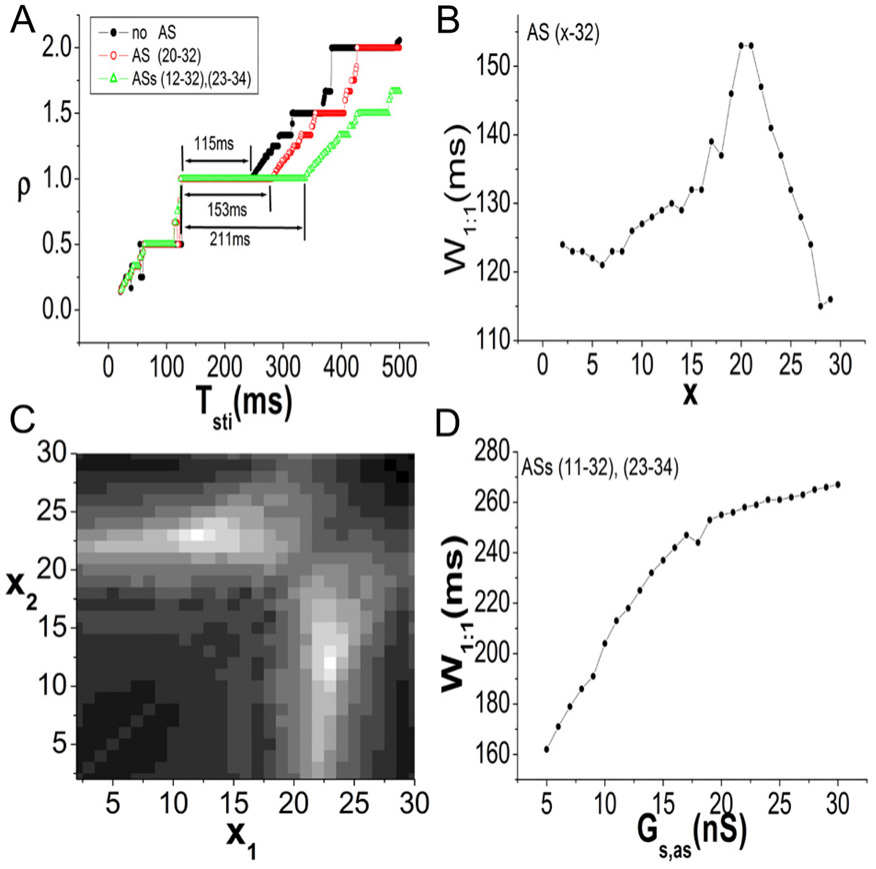
The effect of ASs on the 1:1 range. We fix *G*_*ss*_ = 20 nS and *G*_*sa*_ = 20 nS for all cases. In A-C, *G*_*s*,*as*_ = 10 nS. Periodic stimulations (*T*_*sti*_ is used to denote the period) are delivered to the 29th cell, and data are measured at the 10th SAN cell. A: The “devil stairs” show phase-locking regions. The 1:1 widths of systems without and with AS(s) are indicated in the figure. The 1:1 range is enlarged by AS(s). However, the other *N* : *M* ranges are not as nearly impacted. B: The 1:1 width depends on the linking position *x* of AS (*x*-32). When *x* is varied within the SAN, the greatest enlargement appears at *x* = 21. C: The effect of two ASs (*x*_1_-32) and (*x*_2_-34). As *x*_1_ and *x*_2_ are varied within the SAN tissue, the 1:1 widths are shown in the *x*_1_-*x*_2_ plane in colors ranging from black to white. The lighter the color is, the larger the value. The peak values occur at (*x*_1_ = 23, *x*_2_ = 12) and (*x*_1_ = 12, *x*_2_ = 23). D: The 1:1 width as a function of *G*_*s*,*as*_. Two ASs of (11–32) and (23–34) are added to the system. The values of *G*_*s*,*as*_ are identical for the ASs. As the coupling is increased, the 1:1 width is increased.

The effect of ASs on the phase-locking ranges is shown in Fig. 6A. Interestingly, with AS(s), the 1:1 range is evidently enlarged (the widths are indicated in the figure), but the other phase-locking regions are not as influenced. Note that the alternans rhythm 2:2 is not regarded as 1:1 even though its rotation number is 1. Because of heterogeneity, ASs linking different positions in the SAN cable should yield different lengths of the 1:1 range. We systematically investigate the effects of the linking positions. First, a single AS (*x*-32) is analyzed. The linking position *x* of the left end of the AS is scanned from the central to peripheral ends of the SAN tissue. The width 1:1 as a function of *x* is plotted in Fig. 6B. The AS can always enlarge the 1:1 range. There is a peak at *x* = 21. Second, the case of two ASs (referred to as (*x*_1_-32) and (*x*_2_-34)) is investigated, with *x*_1_ and *x*_2_ being scanned from the central to peripheral ends of the SAN (*x*_1_ ≠ *x*_2_). Fig. 6C shows the 1:1 width in the *x*_1_-*x*_2_ plane. The colors represent the widths, with a lighter color indicating a larger region. The peak values occur at (*x*_1_ = 23, *x*_2_ = 12) and (*x*_1_ = 12, *x*_2_ = 23). Two ASs can enlarge the 1:1 width to a greater extent than one AS (the highest width is 211 ms for two ASs, which is considerably larger than the width of 153 ms for one AS). Consequently, it can be concluded that the system with AS(s) possesses a wider 1:1 range than that without an AS. In particular, when one of the ASs is linked to the positions around the 22nd SAN cell, the enlargement can be very great. In addition to the linking positions, the coupling strengths between the SAN and ASs also influence the 1:1 width. We randomly add two ASs of (11–32) and (23–34) to the system to evaluate the effect. These two ASs have an identical *G*_*s*,*as*_ value. The 1:1 width as a function of *G*_*s*,*as*_ is shown in Fig. 6D. Increasing the SAN-AS coupling can also effectively increase the 1:1 width.

**Fig 7:**
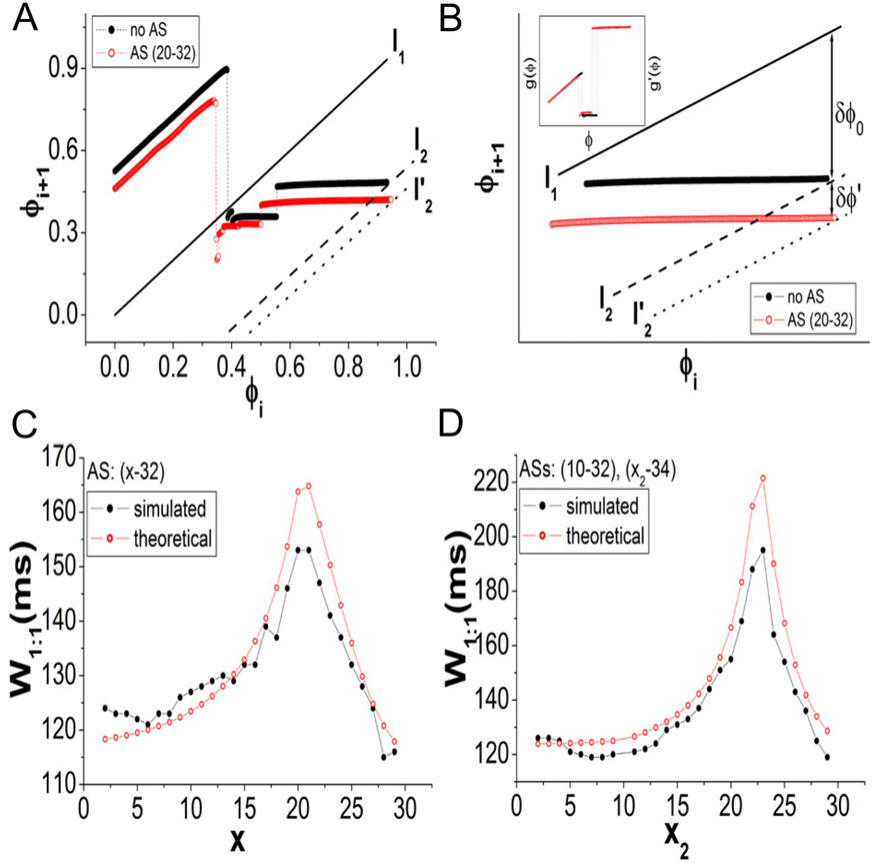
Explanation of the 1:1 enlargement by PRM. *G*_*ss*_ = 20 nS, *G*_*sa*_ = 20 nS and *G*_*s*,*as*_ = 10 nS. A: PRMs without an AS (black solid circles) and with AS (20–32) (red empty circles). *T*_*sti*_ = 129 ms. The solution of the map is determined by the relative positions of the PRM curve and the straight line. Increasing *T*_*sti*_ shifts the PRM upward (see Eq. (6)). Equivalently, the line moves downward relative to the PRM. We show the motion of the line rather than that of the PRMs in the figure. For both the systems with and without an AS, the 1:1 range can roughly be considered to begin at *T*_*sti*_ = 129 ms, and the line crosses the position labeled “l_1_”. When the line reaches the position labeled “l_2_”, corresponding to *T*_*sti*_ = *T*_2_, the 1:1 range ends in the system without an AS. For the system with an AS, the 1:1 range should end at “${\text{l}}_{2}^{\prime }$”. Therefore, the 1:1 range persists longer in the system with an AS. B: The magnification of the flat sections of PRMs in A. *δϕ*_0_ is the difference between l_1_ and l_2_, which determines the 1:1 width without an AS. *δϕ*′ is the discrepancy between the PRMs when *T*_*sti*_ = *T*_2_. *δϕ*_0_ + *δϕ*′ determines the 1:1 width with an AS. The inset shows the phase transition curves of the systems without and with an AS, which approximately coincide, and thus, we obtain *δϕ*′ (see Eq. (10)). C and D: Comparisons between the simulated and theoretical results. The parameters are identical to those used in Figs. 6B and 6C. In D, the simulated results are taken from Fig. 6C by fixing *x*_1_ = 10.

Based on the above findings, we attribute the enlargement of the 1:1 range to the reduction in the automaticity of the SAN, which results from the hyperpolarization due to the ASs. The automaticity of SAN tissue can be evaluated by its intrinsic oscillating period. Therefore, any factor that increases the intrinsic period of the SAN can enlarge the 1:1 range of the system. This hypothesis can be verified using PRM (Eq. (6)). We randomly choose an AS (20–32) as an example. In Fig. 7A, the curve plotted using the black solid circles represents PRM without AS and that using the red empty circles represents PRM with AS (20–32). The data are measured from the stimulated cell (the 29th cell). *T*_*sti*_ = 129 ms, which is the beginning of the 1:1 range of the system without an AS (i.e., the left boundary of the 1:1 range in the *T*_*sti*_ − *ρ* diagram), is used to determine the PRMs. We can also regard *T*_*sti*_ = 129 ms as the approximate beginning of the 1:1 range of the system with an AS. The AS on the PRM shifts the whole curve downward relative to the one without an AS (also causing a slight difference in shape). According to the theory of nonlinear dynamics, the solution of the map is determined by the relative positions of the PRM curve and the straight line representing *ϕ*_*i*+1_ = *ϕ*_*i*_ (labeled “l_1_” in Fig. 7A). If the line intersects the PRM within its flat section, where the slope is approximately 0, 1:1 is stably realized. Therefore, the 1:1 range is determined by the range that encompasses the movement of the intersection point within the flat section. It is known that an increase in *T*_*sti*_ can shift the entire PRM curve upward (see Eq. (6) and Ref. [36]). Equivalently, this means that the line shifts downward relative to the PRM. To avoid drawing a number of PRMs in the figure, we use the motion of the line to describe the dynamics of the PRM. In Fig. 7A, when *T*_*sti*_ = 129 ms, the line crosses the position labeled “l_1_”. As *T*_*sti*_ increases, the line shifts downward. When the position of the dashed line labeled “l_2_” is reached, the 1:1 range of the system without an AS ends. However, for the system with an AS, the 1:1 range should end at the position of the dotted line labeled ${\text{l}}_{2}^{\prime }$. Therefore, the 1:1 range can persist longer in the system with an AS. The other *N* : *M* phase-locking regions are tangentially bifurcated from 1:1. Because the shape of the PRM does not appear to be influenced by the AS, the other *N* : *M* ranges do not appear to be changed by the AS.

Based on the above analysis, a formula for approximating the enlargement of the 1:1 range can be derived. The flat sections of the PRMs in Fig. 7A are drawn with exaggeration in Fig. 7B. The lines l_1_ (solid) and l_2_ (dashed) indicate the beginning and ending states of the 1:1 range for the system without an AS, corresponding to *T*_*sti*_ = *T*_1_ and *T*_*sti*_ = *T*_2_, respectively. Thus, according to Eq. (6), the shifting range between l_1_ and l_2_ in the diagram is

$$\begin{array}{c} \delta \phi_{0}=\frac{T_{2}-T_{1}}{T_{0}}=\frac{W_{1:1}}{T_{0}}, \end{array}$$ (7)

where *T*_0_ is the intrinsic period of the system without an AS and *W*_1:1_ is the width of the 1:1 range. For the system with an AS, the end of the 1:1 range is indicated by ${\text{l}}_{2}^{\prime }$ (dotted), corresponding to $T_{sti}=T_{2}^{\prime }$. Similarly, the shifting range between l_1_ and ${\text{l}}_{2}^{\prime }$ in the diagram is

$$\begin{array}{c} \delta \phi_{0}+\delta \phi^{\prime }=\frac{T_{2}^{\prime }-T_{1}}{T_{0}^{\prime }}=\frac{W_{1:1}^{\prime }}{T_{0}^{\prime }}. \end{array}$$ (8)

Here, $T_{0}^{\prime }$ is the intrinsic period with an AS, which is generally larger than *T*_0_. By substituting Eq. (7) into Eq. (8), the 1:1 range with an AS can be obtained:

$$\begin{array}{c} W_{1:1}^{\prime }=\frac{T_{0}^{\prime }}{T_{0}}W_{1:1}+T_{0}^{\prime }\delta \phi^{\prime }. \end{array}$$ (9)

The quantity *δϕ*′ is further explained as follows.

Initially, when *T*_*sti*_ = 129 ms (the beginning of 1:1), there is an initial discrepancy between the two PRMs (as shown in Fig. 7A). As *T*_*sti*_ is increased, the shifting velocities of PRMs with and without an AS are different (see Eq. (6); the shifting velocities are determined by 1/*T*_0_ and $1/T_{0}^{\prime }$), and thus, the discrepancy between the maps will vary. When the 1:1 range ends at the PRM without an AS at *T*_*sti*_ = *T*_2_, the distance between the two PRMs at this state is *δϕ*′. At *T*_*sti*_ = *T*_2_, we can obtain two values of *ϕ*_*i*+1_ from the PRMs for identical *ϕ*_*i*_, and *δϕ*′ is the difference between the two *ϕ*_*i*+1_ values. The two values of *ϕ*_*i*+1_ can be expressed as:

$$\begin{array}{ccc} \phi_{i+1} & = & \phi_{i}-\Delta \phi_{i}+\frac{T_{2}}{T_{0}}=g\left(\phi_{i}\right)+\frac{T_{2}}{T_{0}}\;\left(\text{without an AS}\right)\\ \phi_{i+1}^{\prime } & = & \phi_{i}-\Delta \phi_{i}^{\prime }+\frac{T_{2}}{T_{0}^{\prime }}=g^{\prime }\left(\phi_{i}\right)+\frac{T_{2}}{T_{0}^{\prime }}\;\left(\text{with an AS}\right). \end{array}$$

*g*(*ϕ*) = *ϕ*_*i*_ − Δ*ϕ*_*i*_ and $g^{\prime }(\phi )=\phi_{i}-\Delta \phi_{i}^{\prime }$ are the so-called phase transition curves [22, 24]. We plot *g*(*ϕ*) and *g*′(*ϕ*) as functions of *ϕ* in the inset of Fig. 7B. We observe that the curves of the systems with and without an AS nearly coincide with each other, i.e., *g*(*ϕ*) ≈ *g*′(*ϕ*). Consequently,

$$\begin{array}{c} \delta \phi^{\prime }=\phi_{i+1}-\phi_{i+1}^{\prime }=\frac{T_{2}}{T_{0}}-\frac{T_{2}}{T_{0}^{\prime }}. \end{array}$$ (10)

By substituting Eq. (10) into Eq. (9), we can finally obtain the formula for estimating $W_{1:1}^{\prime }$:

$$\begin{array}{c} W_{1:1}^{\prime }=\frac{T_{0}^{\prime }}{T_{0}}W_{1:1}+T_{2}\left(\frac{T_{0}^{\prime }}{T_{0}}-1\right). \end{array}$$ (11)

The quantities *T*_0_, *W*_1:1_ and *T*_2_ of the system without an AS can be measured in advance. The intrinsic period $T_{0}^{\prime }$ determined by ASs is the only quantity that is required to estimate the enlarged width of the 1:1 range. The parameters used in the present model are *T*_0_ = 245.86 ms, *W*_1:1_ = 115 ms and *T*_2_ = 243 ms.

Comparisons between the theoretical and simulated results are shown in Figs. 7C and 7D. The parameters are identical to those used in Figs. 6B and 6C. Fig. 7C shows the case for a single AS. In Fig. 7D, the simulated results (black solid circles) are taken from Fig. 6C by fixing *x*_1_ = 10. The theoretical results are qualitatively consistent with the simulated ones. We believe that the quantitative discrepancies primarily result from the assumption that the 1:1 range starts at *T*_*sti*_ = 129 ms for the system with an AS, which is quantitatively imprecise. In fact, there are fine structures near the bifurcation point of 1:1 [22], which render phase-locking behaviors to be very complicated. However, Eq. (11) provides a qualitatively satisfactory estimation. According to Eq. (11), any factor that increases $T_{0}^{\prime }$ can increase the 1:1 range, and an increase in the AS number and coupling strength *G*_*s*,*as*_ can slow the SAN frequency and increase the 1:1 range.

In this subsection, we found that ASs penetrating into the SAN periphery region can enlarge the 1:1 synchronization range. Moreover, a practical formula for approximating the enlargement of the 1:1 range was derived. We conclude that ASs penetrating into the peripheral SAN effectively enlarge the 1:1 phase-locking range of the SAN.

### Limitations

Based on the above results, we can see that the conduction and phase-locking dynamics of SAN-atrium system depend on the following properties: the oscillation of heterogeneous SAN tissue, the hyperpolarization from atrium tissue and strands, and the excitability of atrial cells. The mechanisms are general for coupled oscillatory-excitable systems. Therefore, the above discussions are qualitatively model independent. However, we have to point out that the physiology and geometry of our model are oversimplified, which may yield quantitative deviations from real heart, and lack of some possible complex dynamics. The limitations of the model are discussed as follows.

(1) The SAN model. SAN model used in the present work treats SAN cell as a pure electrical oscillator, while recent experimental results discovered that calcium cycling process in sarcoplasmic reticulum also contributes to the oscillation of SAN cell [38]. The interplay between intracellular calcium concentration and voltage may induce more complex oscillation patterns of SAN tissue (just like that in ventricular tissue [47]).

(2) The atrial cell model. In the present study, we used the LR1 model to represent the atrial cell. Since the focus of the study is on conduction and phase-locking which mainly depend on excitability of the cell, not on other detailed physiological properties, we believe that our conclusions will generally hold true. Nevertheless, we also carried out simulations by substituting the LR1 model by the Earm-Hilemann-Noble atrial cell model [48] and repeated simulations for the main results shown in Fig. 4 and Fig. 6A. The results are shown in the supporting information S1 Text. Using the Earm-Hilemann-Noble model gives rise to almost identical results to those of using the LR1 model.

(3) The 1D cable approach. Although our 1D cable model can reveal the basic pacing and conduction properties of the system, it cannot reveal the complex patterns of arrhythmias in 2D and 3D tissue. Therefore, the overall effects of the ASs should be evaluated in 2D and 3D models, which is a valuable problem for future study.

(4) Other factors. As indicated by Ref. [9], factors such as coupling gradient and fibroblasts also influence the pacing and conduction of SAN-atrium system, which should be considered in the complex model.

In the present stage our work reveals the basic mechanisms of the propagation and phase-locking behaviors in presence of ASs. Experimental verifications of our theoretical results are waited. To better approach the real heart situations, more complexities and modern developed models should be incorporated into the future studies.

## Conclusion

In this paper, the functions of the ASs in terms of interdigitating with and penetrating into the SAN are investigated. The heterogeneity and geometric complexity of the SAN-atrium system are considered. In such a realistic model, the functions of ASs are as follows: (i) atrial strands linking the central SAN play a major role in impulse propagation, because the threshold coupling required to excite the AS is low at the center. (ii) Atrial strands that link the peripheral SAN are the primary ASs responsible for enlarging the 1:1 range, because the automaticity of the SAN is reduced. A practical formula for estimating the enlargement is proposed.

In cardiology there are some problems closely associated to impulse propagation and phase-locking dynamics, e.g., the conduction block between SAN and atrium [49], the parasystole resulted by the interactions between sinus rhythm and ectopic beat [22, 50], and the sinus rhythm modulated by acetylcholine pulses [14]. Furthermore, it is found that the frequency of the SAN cell itself is determined by interplay between voltage clock and internal calcium clock [38], which can also be fundamentally interpreted by phase-locking theories. The present study may provide novel insights into the issues. For example, we hypothesize that the wide adjustable range of stable synchronous beating of SAN-atrium system in vivo may be relevant to the enlarged 1:1 range. We suggest that studies of the above problems should be carried out in realistic cardiac models and experimental tissue considering the functional complexity such as heterogeneity and atrial strands, so that some more interesting and reasonable results may be revealed. Moreover, the results of the present work may be applied to understand and predict the behaviors of general oscillatory-excitable complex systems. Thus we hope that this work may also enrich the fundamental theories of nonlinear dynamics and science of complex systems.

## Supporting Information

S1 Text Comparisons between the LR1 and EHN model. (DOC)
