# Supplementary material for: The Functions of Atrial Strands Interdigitating with and Penetrating into Sinoatrial Node: A Theoretical Study of the Problem
Source: PLoS One. 2015 Mar 24;10(3):e0118623. doi: 10.1371/journal.pone.0118623 (PMC4372425; doi:10.1371/journal.pone.0118623)
Supplement: S1 Text — (DOC) [file pone.0118623.s001.doc]

**Supporting information**

We performed simulations using the Earm-Hilgemann-Noble (EHN) atrial cell model and repeated the simulations shown in Figure 4 and Figure 6A in the paper. The figure compares the results of using the both models.

The parameters of the EHN model are the same as the original ones on <http://www.cellml.org/> (also see Winslow RL et al. (1993) Generation and propagation of ectopic beats induced by spatially localized Na-K pump inhibition in atrial network models. Proc. R. Soc. Lond. B 254: 55-61). The coupling parameters of G_ss_, G_s,as_, G_sa_ and G_aa_ are identical to those used for the LR1 model, except that In panel B G_sa_ is reduced to 8nS to block conduction via the peripheral SAN to estimate the threshold G_s,as_ (10 ns was used for the LR1 model). The reason is that the EHN model is more excitable than the LR1 model.


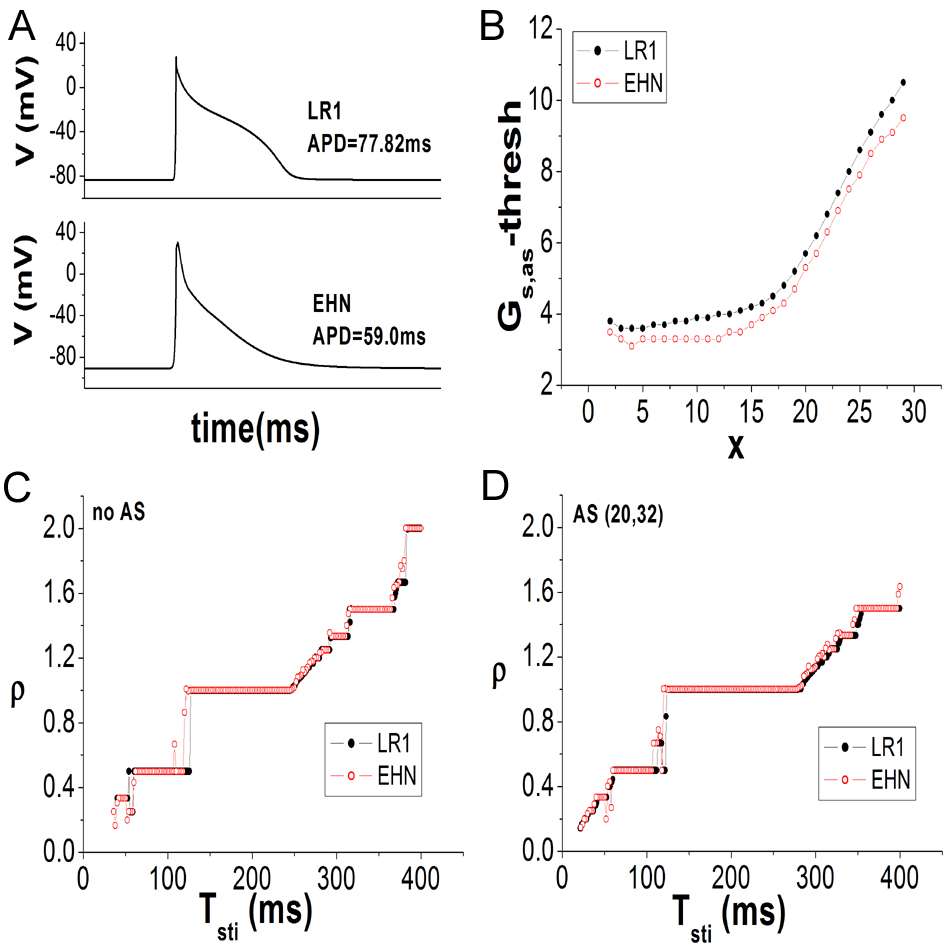


Panel A shows the action potentials of the two models, which are measured from a cell in tissue under identical pacing rate of SAN. Panel B shows the threshold G_s,as_. The values of the EHN model is a little bit smaller than that of the LR1 model because of different excitability of the models. Panels C and D are the devil’s stairs of the systems without AS and with AS (20,32), respectively. Under the same conditions, the phase-locking regions of the two models coincide well with each other.
